# Primary intracranial sarcomas: a clinicopathological investigation

**DOI:** 10.3389/fonc.2023.1195467

**Published:** 2023-06-09

**Authors:** Yu-Xiang Cai, Jin-Sheng Liu, Jian Xu, Yong-Ze He, Huai-Nian Zhang, Su-Fang Tian, Zhi-Qiang Li

**Affiliations:** ^1^ Department of Pathology, Zhongnan Hospital of Wuhan University, Wuhan, China; ^2^ Department of Neurosurgery, Zhongnan Hospital of Wuhan University, Wuhan, China

**Keywords:** primary intracranial sarcomas, case series, pathology, MRI, next-generation sequencing

## Abstract

**Background:**

The purpose of this study is to present a series of primary intracranial sarcomas (PIS), a rare type of tumor of the central nervous system, in order to improve our understanding of the disease. These tumors are heterogeneous and prone to recurrence after resection, exhibiting a high mortality rate. As PIS has yet to be understood and studied on a large scale, it is vital for further evaluation and research.

**Methods:**

Our study included 14 cases of PIS. The patients’ clinical, pathological, and imaging features were retrospectively analyzed. Additionally, targeted DNA next-generation sequencing (NGS) was applied for the 481-gene panel to detect gene mutations.

**Results:**

The average age for PIS patients was 31.4 years. Headache (7, 50.0%) was the most common symptom leading to the hospital visit. Twelve cases had PIS located in the supratentorial area and two in the cerebellopontine angle region. The maximum tumor diameter ranged from 19.0 mm to 130.0 mm, with an average diameter of 50.3 mm. Pathological types of tumors were heterogeneous, with chondrosarcoma being the most common, followed by fibrosarcoma. Eight of the 10 PIS cases that underwent MRI scanning showed gadolinium enhancement; 7 of these cases were heterogeneous, and 1 of them was garland-like. Targeted sequencing was performed in two cases and identified mutations in genes such as NRAS, PIK3CA, BAP1, KDR, BLM, PBRM1, TOP2A, DUSP2, and CNV deletions of SMARCB1. Additionally, the SH3BP5::RAF1 fusion gene was also detected. Of the 14 patients, 9 underwent a gross total resection (GTR), and 5 chose subtotal resection. Patients who underwent GTR displayed a trend toward superior survival. Among the 11 patients with available follow-up information, one had developed lung metastases, three had died, and eight were alive.

**Conclusion:**

PIS is extremely rare compared to extracranial soft sarcomas. The most common histological type of intracranial sarcoma (IS) is chondrosarcoma. Patients who underwent GTR of these lesions showed improved survival rates. Recent advancements in NGS aided in the identification of diagnostic and therapeutic PIS-relevant targets.

## 1 Introduction

Primary intracranial sarcomas (PIS) are a rare group of heterogeneous tumors (1), with their prevalence ranging from 0.1% to 4.3% across the literature (2). These tumors probably have the origin of multipotent primitive mesenchymal cells within leptomeninges or dura (1, 3–5). The ionizing radiation is the only evident associated risk factor (4, 5). Combining clinical and imaging data with a histopathological examination of PIS aids in its diagnosis (1).

The underlying mechanisms of PIS remain unclear due to its rarity and heterogeneity. In pediatric sarcomas, there is significant activation of signaling pathways, such as angiogenesis as well as receptor tyrosine kinases (RTKs), associated with tumor survival (6–9). The platelet growth factor receptor (PDGFR), as an RTK, shows overexpression within Ewing sarcoma, rhabdomyosarcoma, and osteosarcoma (OS) (9–12). Its involvement in tumorigenesis bears considerable implications in terms of prognosis and treatment. PIS does not have an accurate approach to its treatment established to date. A combination of surgical and adjuvant therapies is typically utilized in PIS management, whereas radiation therapy is a common approach for local disease control (9, 13, 14). Physicians can better comprehend the molecular characteristics of the disease and identify potential treatments when next-generation sequencing (NGS) and other molecular biology techniques are applied in clinical practice. Moreover, this high-throughput approach has the potential to identify critical information that could lead to changes in the diagnosis (15).

The knowledge concerning clinicopathological features of PIS is deficient as fewer studies have been conducted on PIS. Thus, an investigation of 14 PIS cases was conducted to comprehend their behavior and prognosis better. Furthermore, targeted sequencing was carried out on some of the PIS cases to obtain diagnostic and therapeutic insight.

## 2 Materials and methods

### 2.1 Selection of patients

In this study, 14 cases of PIS were retrospectively collected from the archives of Zhongnan Hospital of Wuhan University. Cases of malignant meningioma, gliosarcoma, malignant solitary fibrous tumor, and intracranial metastatic sarcoma were excluded from the study. Clinical data obtained from the patients included their sex, age, location, clinical manifestations, and radiologic appearances. Clinical courses of patients were tracked retrospectively. All cases were reviewed by two pathologists (ST, YC) who assessed their pathological features. This study was approved by the Institutional Review Board at the Zhongnan Hospital of Wuhan University (approval code: 2022007K).

### 2.2 Histopathology and immunohistochemistry

The specimens were fixed with 10% neutral formalin solution, routinely embedded in paraffin, 4μm thick sections were stained with haematoxylin and eosin-stain and immunohistochemistry. The following antibodies were performed using automated instruments CK (AE1/AE3, ZSGB-BIO, prediluted), EMA (GP14, ZSGB-BIO, prediluted), Bcl-2 (EP36, ZSGB-BIO, prediluted), SMA (UMAB237, ZSGB-BIO, prediluted), Dsmin (OTI4A8, ZSGB-BIO, prediluted), CD34 (QBEnd10, DAKO, prediluted), MDM2 (1E6&17B3, ZSGB-BIO, prediluted), CD99 (12E7, DAKO, prediluted), SATB2 (OTI5H7, ZSGB-BIO, prediluted), TLE1 (UMAB253, ZSGB-BIO, prediluted), SSTR-2 (EP149, ZSGB-BIO, prediluted), ALK (OTI1H7, ZSGB-BIO, prediluted), S-100 (15E2E2 + 4C4.9, ZSGB-BIO, prediluted), IDH1 (H09, ZSGB-BIO, prediluted), Myogenin (EP162, ZSGB-BIO, prediluted), MyoD1(EP212, ZSGB-BIO, prediluted), CyclinD1(SA38-08, ZSGB-BIO, prediluted), CD68(KP1, ZSGB-BIO, prediluted), SMARCB1 (OTIR4G9, ZSGB-BIO, prediluted), STAT6 (EP325, ZSGB-BIO, prediluted), GFAP (EP13, ZSGB-BIO, prediluted), Olig-2 (EP112, ZSGB-BIO, prediluted), Ki-67 (MIB-1, ZSGB-BIO, prediluted).

### 2.3 Targeted next-generation DNA sequencing analysis

DNA was extracted from five 10-µm sections from formalin-fixed paraffin-embedded (FFPE) tissue, with the help of QIAamp DNA FFPE Kit (QIAGEN, Valencia, CA, USA) in line with specific protocols. DNA quality was assessed through spectrophotometry, and optical density (OD) values were detected at 230, 260, and 280 nm. Qubit 3.0 software was employed for DNA quantification. Library preparation was carried out according to the previous description (16). Through the adoption of xGen Lockdown Hybridization and Wash Reagents Kit (Integrated DNA Technologies), GeneseeqOne™ bone tumor gene panel (481-cancer-relevant genes, Geneseeq Technology Inc.) was utilized for the enrichment of target based on hybridization. Libraries were captured through Dynabeads M-270 (Life Technologies), which were later subjected to amplification within the KAPA HiFi HotStart ReadyMix (KAPA Biosystems) as well as quantified with KAPA Library Quantification kit (KAPA Biosystems) employing the qPCR. Later, the target enriched libraries were sequenced with the help of the NovaSeq4000 platform (Illumina), with pair-end reads of 2 × 150bp. The sequencing data were demultiplexed by utilizing software bcl2fastq (v2.19), whereas Trimmomatic was utilized for the analysis (17). For removing N-bases or those bases with low-quality (quality<15), Burrows-Wheeler Aligner was used to map sequencing data to reference the hg19 genome (Human Genome version 19) (18). Picard (https://broadinstitute.github.io/picard/) was utilized to remove PCR duplicates. Indels-surrounding local realignments along with base-quality reassurance were conducted using Genome Analysis Toolkit (GATK) (19). At least three distinct mutant reads and a mutant allele frequency (MAF) threshold of 0.5% were used in HaplotypeCaller/UnifiedGenotyper in GATK and VarScan2 (20) that were later applied in calling indels and SNPs. Common variants were removed using dbSNP as well as the 1000 Genome project. As for germline mutations, they were compared against whole blood controls in the cases of filtration. The in-house list of relapsed sequencing errors was obtained, based on more than 10,000 healthy subjects using an identical sequencing platform, and was used to filter the obtained somatic variants. For identifying gene fusions, FACTERA (6) was adopted, whereas ADTEx (21) was utilized for analyzing copy number variations (CNVs). The threshold log2 ratio was deemed to be 2.0 for copy number gain in the tissues, while in the whole samples, it was deemed to be 0.67 for copy number loss. Thresholds based on prior assay verification were determined through the droplet digital PCR (ddPCR)-detected absolute CNVs. FACETS (22) was employed for analyzing allele-specific CNVs, and upon the drift threshold of 0.2, unstable joint segments were determined. To determine the chromosomal instability (CIN) proportion, the drifted segment size was divided by the overall segment size.

## 3 Results

### 3.1 Clinical features and demographic information of the patients

The clinical features of the cases are described in **Table 1**. There were 14 patients included in the study, 8 male and 6 female, with a mean age of 31.4 years (median 28, range 3–71). The most common symptom of a hospital visit was headache (7), (50.0%), dizziness ranks the second most common symptom (4), (28.6%). Other presenting symptoms inclued twitching of extremities, blindness, numbness, and hearing loss. The tumor was located supratentorial are in 12 cases (85.1%), and 2 case situated in cerebellopontine angle. The maximum diameter tumor calculated based on radiographic examination ranged from 19.0 to 130.0 mm, with a average of 50.3 mm.

**Table 1:** Clinical characteristics of PIS patients.

Case no.	Sex	Age	Location	Manifestations	Tumor size (mm)
1	M	4	right frontal lobe	headache with vomiting	52
2	M	3	left temporo-occipital lobe	twitching of extremities	88
3	M	34	right frontal lobe	numbness in the left limb	19
4	M	34	left temporo-parietal lobe	dizziness and weakness	42
5	M	26	right parieto-occipital lobe	headache with dizziness	55
6	F	27	left parieto-occipital lobe	headaches with dizziness	40
7	M	27	left maxillofacial and fronto-temporal-parietal region	post-operative of “meningiom”, left temporal scalp splitting and bleeding	130
8	F	71	inside and outside of the left frontal cranial plate	headache	30
9	F	42	clivus	blurred vision with occasional headaches	30
10	M	28	right occipital region	right occipital mass	45
11	F	43	right cerebellopontine angle region	hearing loss on the right side with tinnitus	48
12	M	28	left cerebellopontine angle region	hearing loss in left ear	70
13	F	27	right parasellar	dizziness and headache	30
14	F	46	clivus	headache with double vision	25

### 3.2 Pathological findings and genomic features

**Figure 1:**
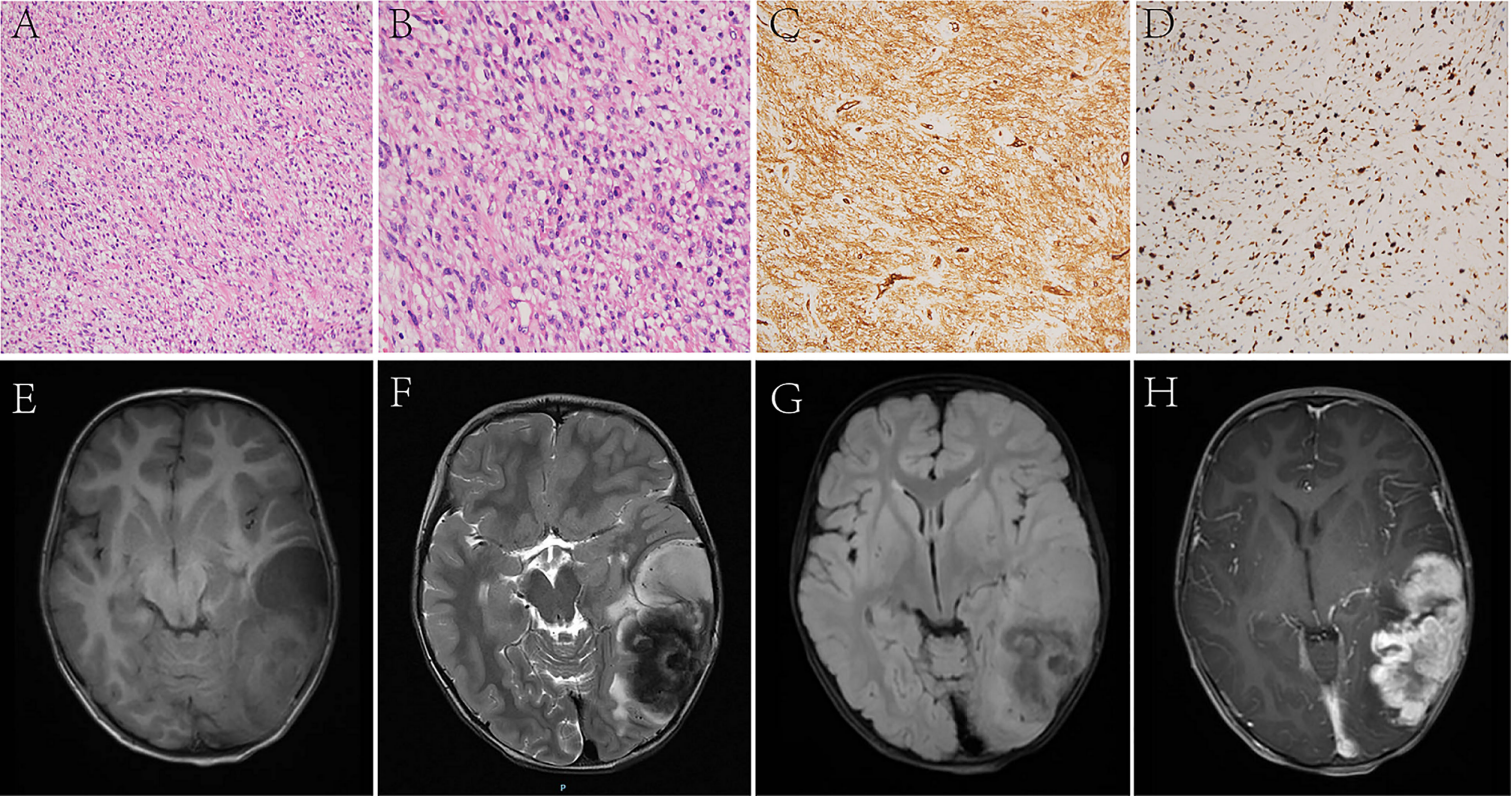
In Case 2, the tumor cells are long spindles, relatively uniform in size, and arranged in bundles **(A, B)**. The IHC analysis displays positiveness for CD34 **(C)**, and the Ki-67 LI is about 30% **(D)**. MRI shows that the tumor is located in the left temporo-occipital lobe, a solid-cystic irregular appearance, T1WI **(E)**, T2WI **(F)**, and T2 FLAIR **(G)** shows heterogeneous intensity, contrast-enhanced T1WI **(H)** shows garland-like enhancement.

**Figure 2:**
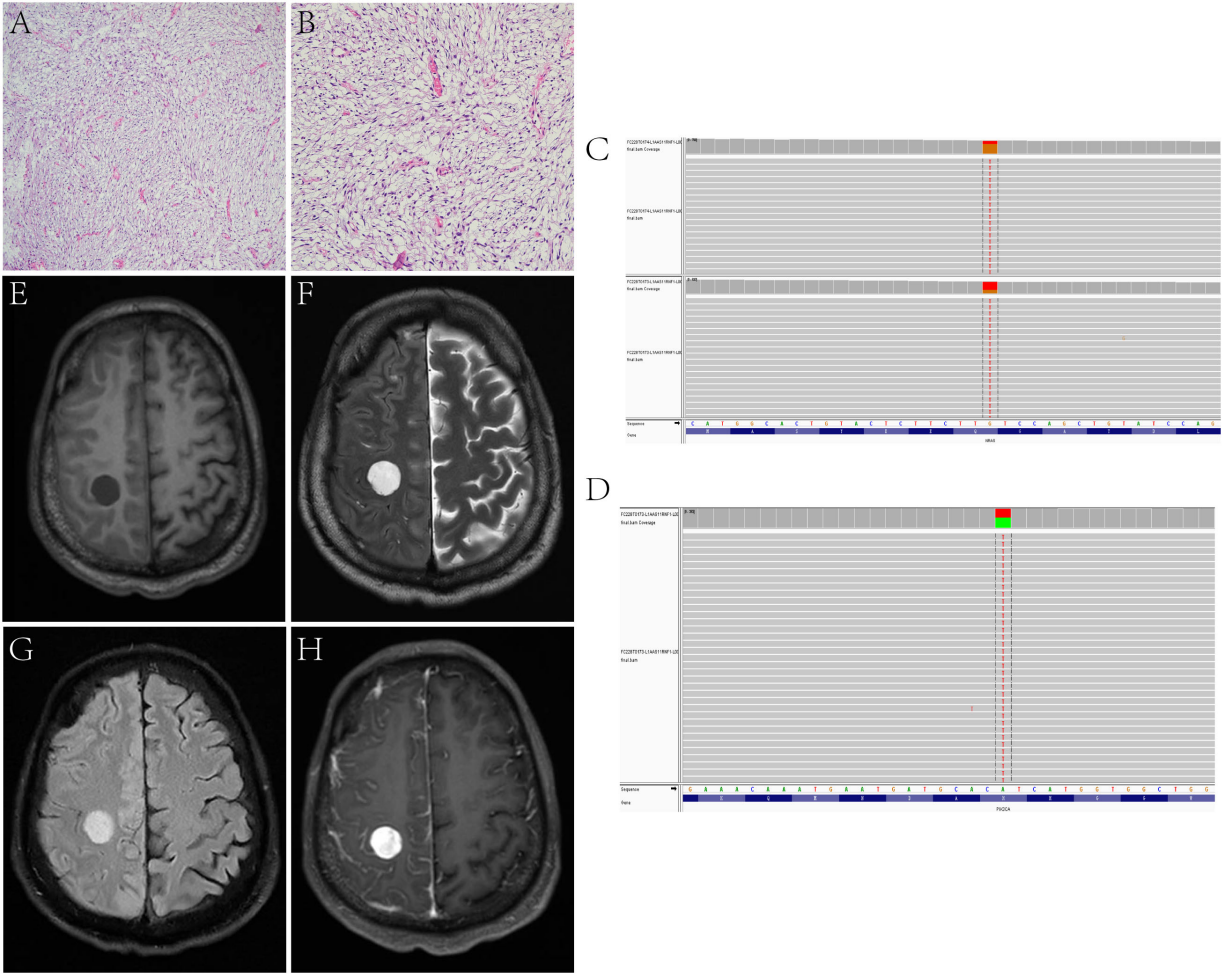
In Case 3, the tumor cells are fusiform, moderately dense, with obviously myxoid stroma and lengthened, curvilinear, thin-walled blood vessels **(A, B)**. Target NGS sequencing finds the mutation of NRAS **(C)** and PIK3CA **(D)**. MRI reveals that a sub-round solid lesion is located in the right frontal lobe, T1-weight imaging (T1WI) **(E)** shows hypointensity, T2-weight imaging (T2WI) **(F)** and T2 Fluid Attenuated Inversion Recovery (T2 FLAIR) **(G)** shows hyperintensity, and contrast-enhanced T1WI **(H)** shows inhomogeneous enhancement.

**Figure 3:**
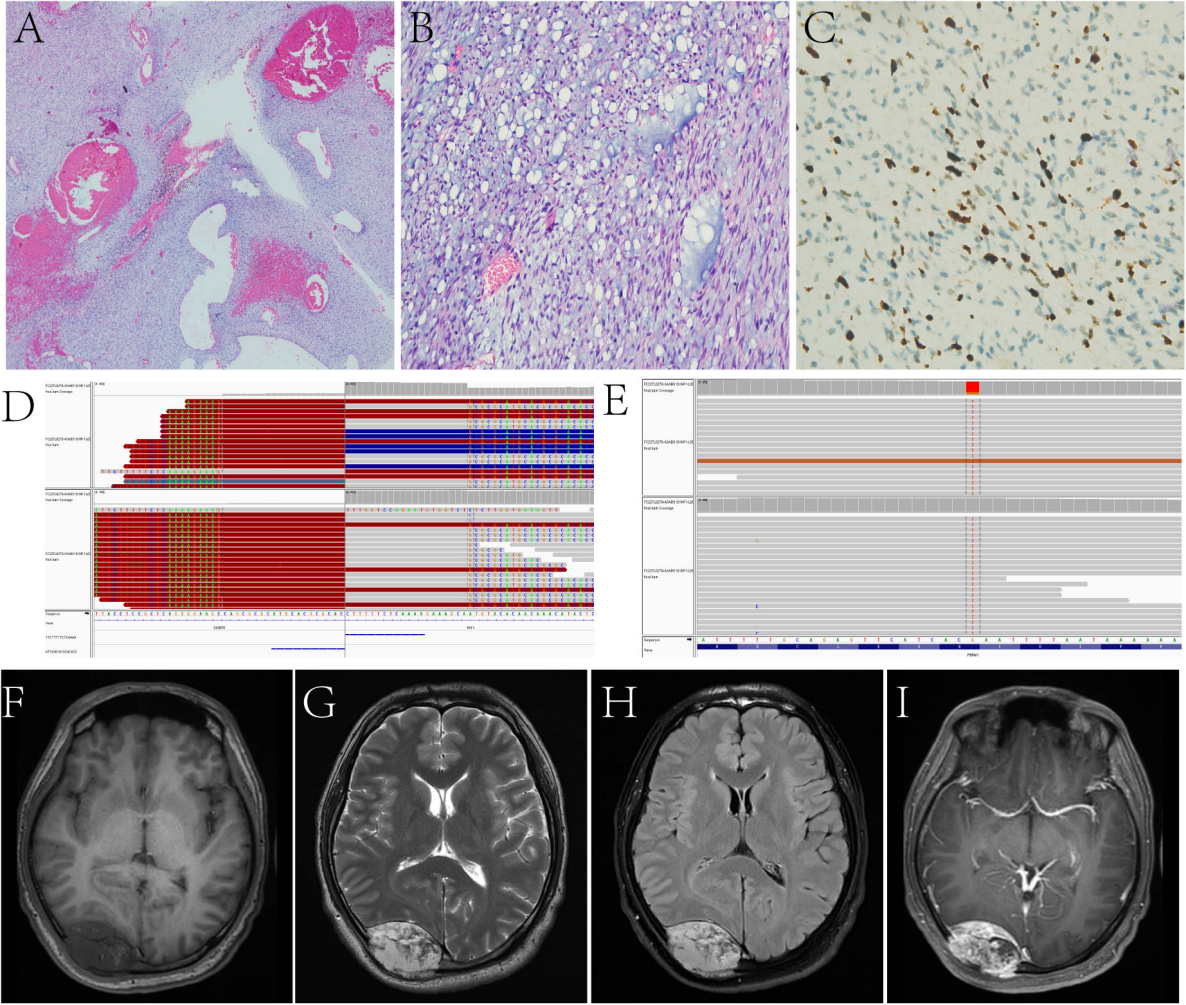
In Case 10, the tumor is arranged in bundles, the myxoid stroma is seen between the tumor cells, and dilated blood vessels are seen inside the tumor **(A, B)**. Ki-67 LI is about 10% **(C)**. Target NGS sequencing finds the SH3BP5::RAF1 gene fusion **(D)** and the mutation of PBRM1 **(E)**. A fusiform solid lesion is sited in the right occipital region and can be seen in the extradural area and invades the skull, T1WI **(F)** shows hypointensity, T2WI **(G)** and T2 FLAIR **(H)** shows hyperintensity, and contrast-enhanced T1WI **(I)** shows inhomogeneous enhancement.

AS showed in **Table 2**, the histological heterogeneity is evident. According to the WHO classification of soft tissue tumors, histologic types were grouped as follows: adult fibrosarcoma [case 1, 2 (**Figure 1**)], myxofibrosarcoma [case 3 (**Figure 2**)], undifferentiated pleomorphic sarcoma (case 4), undifferentiated round cell sarcoma (case 5), leiomyosarcoma (case 6), extraskeletal myxoid chondrosarcoma (case 7), and angiosarcoma (case 8), mesenchymal chondrosarcoma (case 9), undifferentiated spindle cell sarcoma [case 10 (**Figure 3**)], and chondrosarcoma (case 11-14). And there is 10 low-grade lesion, which corresponded to the French Federation of Cancer Centers Sarcoma Group (FNCLCC) system grades 1 and 2. And 4 high-grade lesions, which corresponded to the FNCLCC system grades 3 (2, 23). NGS sequencing was applied in 2 cases (case 3, 10). In case 3, tumor cells are fusiform, moderately dense, with obviously myxoid stroma and elongated, curvilinear, thin-walled blood vessels, tumor cell with ill-defined, slightly eosinophilic cytoplasm and atypical, enlarged, hyperchromatic nuclei. Immunohistochemical revealed MDM-2, SATB2 were positive, GFAP, Olig-2, CD34, Desmin, SMA, S-100, SSTR2, EMA, Myogenin, MyoD1 were negative, and the Ki-67 LI (labeling index) was approximately 20%. Targeted NGS was performed with the resected specimen of case 3, and several key gene mutations were identified including NRAS p.Q61K missense mutation (exon 3), PIK3CA p.H1047L missense mutation (exon 20), BAP1 p.S325F missense mutation (exon 11), KDR p.D1046E missense mutation (exon 23), and BLM p.H651Qfs*4 frame shift mutation (exon 8). In case 10, the tumor cells were short spindle-shaped with round or ovoid nuclei and pale eosinophilic cytoplasm. The tumor was arranged in fascicular and myxoid stroma was seen between the tumor cells, and dilated blood vessels were seen inside the tumor. The tumor cells expressed SATB2, Bcl-2, TLE1, SMARCB1, but not CK, EMA, CD34, SMA, Desmin, S-100, CD99, STAT6, MyoD1, Myogenin, and Ki-67 LI is about 10%. NGS identified PBRM1 p.R876S missense mutation (exon 18), TOP2A p.P371L missense mutation (exon 10), DUSP2 p.C39* nonsense mutation (exon 1), CNV deletions of SMARCB1, and SH3BP5::RAF1 fusion gene. The immunohistochemical expression of PIS is shown in **Table 2**.

**Table 2:** The pathological characteristics of PIS patients.

Case no.	Dianosis	Grade	CK	SMA	Desmin	CD34	CD99	SATB2	TLE1	MDM2	SSTR-2	S-100	Myogenin	Ki-67 (%)	NGS
1	adult fibrosarcoma	G1	–	focal+	–	–	NA	NA	NA	NA	–	–	–	30	NA
2	adult fibrosarcoma	G1	–	–	–	+	–			NA	–	–	–	20	ETV::NTRK3 (-)
3	myxofibrosarcoma	G2	–	–	–	–	–	+	+	+	–	–	–	25	Mutation of NRAS, PIK3CA, BAP1, KDR, BLMT
4	undifferentiated pleomorphic sarcoma	G3	–	–	–	–	–			NA	–	–	–	30	NA
5	undifferentiated round cell sarcoma	G3	–	–	–	–	+	NA	NA	–	–	–	–	85	NA
6	leiomyosarcoma	G3	–	+	+	NA	NA	NA	NA	NA	–	–	–	50	NA
7	extraskeletal myxoid chondrosarcoma	G3	–	–	–	–	NA	NA	NA	NA	NA	–	–	50	NA
8	angiosarcoma	G2	–	NA	NA	+	NA	NA	NA	NA	–	NA	NA	30	NA
9	mesenchymal chondrosarcoma	G2	NA	NA	NA	–	NA	–	NA	NA	NA	NA	NA	40	NA
10	undifferentiated spindle cell sarcoma	G1	–	–	–	–	–	+	+	NA	+	–	–	10	Mutation of PBRM1, TOP2A, DUSP2 CNV deletions of SMAR SH3BP5::RAF1 gene fusion
11	chondrosarcoma	G1	–	–	–	–	NA	NA	NA	NA	+	+	NA	2	NA
12	chondrosarcoma	G1	NA	NA	NA	NA	NA	NA	NA	NA	NA	NA	NA	NA	NA
13	chondrosarcoma	G1	NA	NA	NA	NA	NA	NA	NA	NA	NA	NA	NA	NA	NA
14	chondrosarcoma	G1	–	–	–	–	NA	NA	NA	NA	NA	NA	NA	3	NA

### 3.3 Radiological findings

In the present retrospective study, radiological data were missing for some patients. Among the 10 patients who underwent an MRI scan, the lesions showed hypointensity signals in 6 cases (60.0%), heterogeneous intensity signals in 3 cases (30.0%), and an isointensity signal in 1 case (10.0%) in T1WI. The lesions in 6 cases (60.0%) showed hyperintensity signals, 3 cases (30.0%) showed heterogeneous intensity signals, and 1 case (10.0%) showed an isointensity signal in T2WI. Moreover, 6 cases (60.0%) showed hyperintensity signals, 3 cases (30.0%) showed heterogeneous intensity signals, and 1 case (10.0%) showed an isointensity signal in T2FLAIR. Ten cases accepted the examination of enhanced MRI and showed gadolinium enhancement of the lesion in 8 cases, including 7 heterogeneous enhancements and 1 ring-like enhancement, and they were non-enhanced in 2 cases. In two patients, there was a large band of edema surrounding the lesion (Cases 2, 6). Three out of five patients with brain parenchymal tumors were examined by magnetic resonance spectroscopy (MRS). Two of them (Cases 2, 5) exhibited an elevated Cho peak whereas a reduced NAA peak, and one (Case 9) showed a normal Cho peak whereas a reduced NAA peak in the tumor region. This suggests signs of malignancy in the tumors. The remaining radiological features are detailed in **Table 3**.

**Table 3:** Radiological features of the PIS patients.

Case no.	CT	T1WI	T2WI	T2FLAIR	Enhancement	Other MRI features	Pre-operative imaging diagnosis
1	mixed density	NA	NA	NA	NA	NA	teratoma
2	NA	heterogeneous intensity	heterogeneous intensity	heterogeneous intensity	Garland-like enhancement	intradural, solid-cystic with surrounging edema	glioblastoma
3	slightly low-density	hypointensity	hyperintensity	hyperintensity	inhomogeneous enhancement	intradural, solid	hemangioblastoma
4	low-density	NA	NA	NA	NA	NA	lymphoma
5	NA	heterogeneous intensity	heterogeneous intensity	heterogeneous intensity	inhomogeneous enhancement	intradural, cystic, hemorrhage	glioblastoma
6	NA	heterogeneous intensity	heterogeneous intensity	heterogeneous intensity	inhomogeneous enhancement	intradural, cystic with surround edema, hemorrhage	glioma
7	high-density	NA	NA	NA	NA	NA	meningeoma
8	NA	hypointensity	hyperintensity	hyperintensity	non-enhanced	extradural, inner and outer the skull, skull involvement, solid,	meningeoma
9	slightly high-density	isointensity	isointensity	isointensity	inhomogeneous enhancement	extradural, skull involvement, solid,	Chondrosa rcoma
10	low-density	hypointensity	hyperintensity	hyperintensity	inhomogeneous enhancement	extradural, skull involvement, solid,	chondrosarcoma
11	low-density	hypointensity	hyperintensity	hyperintensity	non-enhanced	intradural, cystic,	acoustic neuroma
12	low-density	NA	NA	NA	NA	none	osteochondroma
13	NA	hypointensity	hyperintensity	hyperintensity	inhomogeneous enhancement	extradural, solid-cystic	meningeoma
14	NA	hypointensity	hyperintensity	hyperintensity	inhomogeneous enhancement	extradural, skull involvement, solid,	chordoma

### 3.4 Surgical information

According to tumor size and location, different incisions as well as surgical approaches were used, such as horseshoe-shaped incision, pterional approach, suboccipital retrosigmoid approach, infratemporal approach, transsphenoidal approach, etc. The character and color of the tumors were different; most of them were fish-meat-like with abundant blood supply. The tumor resection extent was assessed in line with surgical records as well as post-surgery imaging examinations. The details have been described in **Table 4**.

**Table 4:** Surgical information of the PIS patients.

Case no.	Surgical incision/approach	Circumstances of resection	Tumor character/color	Blood supply condition
1	None	subtotal resection	None	None
2	Left temporooccipital region horseshoe-shaped incision	gross total resection	Gray-white	Moderate
3	Right frontoparietal lobe straight incision	gross total resection	furvous	Abundant
4	Left temporal region horseshoe-shaped incision	subtotal resection	Fish-meat-like structure	Moderate
5	None	subtotal resection	None	None
6	Left parietal region horseshoe-shaped incision	gross total resection	Brown-yellow	Abundant
7	Left temporal-parietal region horseshoe-shaped incision	gross total resection	Grey-brown; fish-meat-like structure	Abundant
8	Right frontal region horseshoe-shaped incision	gross total resection	Fish-meat-like structure	Abundant
9	Trans-nasal sphenoidal approach	subtotal resection	unknown	unknown
10	Right temporooccipital region horseshoe-shaped incision	gross total resection	White jelly-like structure	unknown
11	Right suboccipital retrosigmoid approach	gross total resection	Gray-white	Moderate
12	Left suboccipital retrosigmoid approach	subtotal resection	Hard texture and contain a lot of bony structures	Abundant
13	Right pterional approach	gross total resection	yellow	Less
14	Trans-nasal sphenoidal approach	gross total resection	Gray-white	unknown

### 3.5 Adjutant therapy and follow-up

A total of 9 patients received radiotherapy out of the 11 patients with available follow-up information. Only three patients received postoperative chemotherapy. In four patients, tumor recurrence was observed, 1 patient (Case 2) received reoperation, 1 patient (Case 9) received palliative care due to cachexy, and the other patient (Case 7) chose radiotherapy. One patient had lung metastases based on imaging (Case 9). After follow-up, 3 patients died of tumor between 3 months and 15 months, 8 patients were alive. The details can be found in **Table 5**.

**Table 5:** Adjuvant therapy and follow-up information for the PIS patients whose follow-up information was available.

Case no.	Radiotherapy	Chemotherapy	Recurrence/metastasis	Survival (months)
2	Yes, PTV-GTV:50Gy/25F, PTV-CTV:45Gy/25F	No	Yes, 3 month/No	Alive, 12
3	Yes	No	No/No	Alive, 4
4	Yes	Yes	Yes, 5 months/No	Dead,15
5	Yes	Yes	No/No	Dead, 13
7	Yes*	No	Yes, 20 days/No	Dead, 3
9	Yes, PTV-GTV: 54/27F	Yes, IE	Yes, 2 months/Yes, lung	Alive, 7
10	Yes, PTV-GTV: 54Gy/18F	No	No/No	Alive, 5
11	No	No	No/No	Alive, 9
12	Yes	No	No/No	Alive, 24
13	No	No	No/No	Alive, 38
14	Yes, PTV-GTV: 45/25F	No	No/No	Alive, 29

## 4 Discussion

Owing to the rarity and fewer studies conducted on primary intracranial sarcomas (PIS), there exists a lack of understanding concerning their etiology, pathogenesis, and susceptibility factors leading to potential diagnostic challenges and mistreatment (2). Hence, to shed light on the same, the present study was conducted on 14 PIS cases to summarize their clinical and pathological information on PIS.

PIS can affect individuals of all ages; a PIS study based on all age groups indicated that the age at diagnosis ranged from 3 to 63 years (median, 28 years) (2). In the present study, 2 juvenile patients and 12 adult patients were investigated, with an average age of 31 years (median 28 years, range 3–71 years). The majority of the study cases were younger than 50 years, suggesting that PIS might probably take place among young patients, thus, indicating that more attention should be paid to the trend of incidence age. A total of 8 originated from the brain parenchyma, while the remaining 4 originated in the skull or dura mater in our cases. Tumor located supratentorial in 12 cases (85.1%), and other 2 case situated in cerebellopontine angle. Previous reports have shown PIS can be found in the parietal (17.9%), frontal (14.1%), and temporal (14.1%) lobes most common (1, 2, 24). In the present study, the frontal and parietal lobes were the most frequently affected areas; other locations included the temporal lobe, occipital lobe, clivus, cerebellopontine angle region, and parasellar region.

The location of the tumor and its occupying effect are the primary determinants for the clinical symptoms of PIS, often resulting in nonspecific symptoms. In this study, the most common symptoms consisted of headache, dizziness, twitching of extremities, blindness, numbness, and hearing loss. Other symptoms, including memory loss, progressive dementia, sensorimotor dysfunction, and seizures, were also reported (2). In contrast to glial tumors, which tend to stay within the central nervous system, PIS tend to spread beyond it. As a result, up to 40% of individuals with PIS may experience metastatic disease, which is associated with a poor prognosis (13, 14). In the present study, one case displayed imaging evidence of metastasis, thus, highlighting the importance of conducting thorough imaging inspections during the perioperative and follow-up periods.

The PIS management options are akin to those for other CNS tumors and include surgical resection, chemotherapy, and radiotherapy (19–22). Surgery plays a crucial role in both diagnosis and treatment due to its ability to identify molecular and histological changes that are significant in guiding adjuvant therapies (25). In the study, 9 patients achieved gross total resection, and 5 patients underwent subtotal resection. Since PIS tend to be highly aggressive, complete removal of the affected tissue may not be attainable. Therefore, postoperative radiotherapy and/or systemic chemotherapy are necessary to ensure effective treatment (2, 24). In patients with metastatic disease, craniospinal irradiation is deemed a beneficial therapeutic choice (9).

Although various therapies are administered, PIS tends to exhibit lower rates of local tumor control and overall survival in comparison to extracranial soft tissue sarcomas (4, 26). In our study, 4 patients relapsed, 1 patient developed pulmonary metastasis, 3 patients died from the tumor, and 8 patients survived. Previous literature regarding CNS sarcoma recurrence and mortality rates after initial management revealed discouraging results, with 26.9% of patients encountering disease recurrence and 76.9% dying due to disease progression (1). Compared with the literature, we reported a lower recurrence and mortality rate of PIS, which may be related to the shorter follow-up period of some cases. Various pathological subtypes of PIS may display varied prognosis. El Ghatany et al. observed that the survival duration was reduced among patients without GTR, those with metastatic disease, and individuals diagnosed before 1 year of age (13, 14). In our series, patients who had GTR of the tumor had a trend towards superior survival. **Tables 4** and **5** summarizes the clinical characteristics and outcomes.

Based on an analysis conducted by Haider et al. on PIS, fibrosarcoma (16.7%), synovial sarcoma (12.8%), and extraosseous mesenchymal chondrosarcoma (11.5%) were identified as the most frequent histological types (1). The present study, however, revealed chondrosarcoma to be the most prevalent, which was possibly affected by the inclusion criteria of the study; moreover, Haider et al. did not include intracranial skull-based chondrosarcoma in their analysis.

The present research identified interesting molecular alterations in two patients through target next-generation sequencing (NGS). Mutation of PIK3CA and NRAS, the key molecules in the common abnormal pathways (MAPK and PI3K/AKT/mTOR pathways) within cancers, were found in case 3. PIK3CA gene mutations lead to PI3K pathway kinase hyperactivity, causing abnormal cell growth (27–29). Furthermore, these mutations are related to anti-cancer therapeutic efficacy and patient survival (30). The NRAS gene mutations are mostly related to hematopoietic cancers, bladder cancers, and melanomas (31). The activation of the PI3K/Akt/mTOR pathway has been found to be associated with the histologic malignancy and tumor progression of myxofibrosarcoma, and the PI3K/Akt/mTOR pathway has the potential to be a therapeutic target for primary and recurrent myxofibrosarcomas, but no PIK3CA mutation was found in their study (32). NGS identified alterations in genes associated with the SWI/SNF chromatin-remodeling complexes, specifically PBRM1 mutation and CNV deletions of SMARCB1 were found in case 3. The PBRM1 gene activates p21 expression and suppresses cell growth (33). This gene is also necessary for cell aging and DNA double-strand breaks-mediated transcriptional silencing, which enhances DNA damage repair; the inability to perform these functions may be due to mutants of PBRM1 (34). As the tumor suppressor, the SMARCB1 gene is related to chromatin remodeling and is an important part of the SWI/SNF chromatin-remodeling complex (35). Upon SMARCB1 deficiency, SWI/SNF complex cannot bind to the target, causing extensive transcriptional dysregulation. In case 10, the copy number of SMARCB1 was found to be 1.0, suggesting the possibility of heterozygous deletion. IHC also revealed that SMARCB1 expression was not defective, suggesting that it may not be the driver gene for this tumorigenesis. SH3BP5::RAF1, a new fusion gene, was first reported in the present study. Since there is only one case, its function and diagnostic value for this tumor still needs further study.

## 5 Conclusion

In conclusion, PIS is extremely rare compared to extracranially occurring soft sarcomas. Intracranial chondrosarcoma usually originates from the skull, different inclusion criteria led to differences in their incidence. Other type of PIS, including adult fibrosarcoma, myxofibrosarcoma, and undifferentiated sarcoma, which have no exact direction of differentiation. Patients with GTR have a higher survival rate. NGS assay can help identify diagnostic and therapeutically relevant targets, but further clinical studies are needed for personalized therapies and target treatment.

## Data availability statement

The datasets presented in this study can be found in online repositories. The names of the repository/repositories and accession number(s) can be found below: https://www.ncbi.nlm.nih.gov/bioproject/PRJNA978484.

## Ethics statement

The studies involving human participants were reviewed and approved by the Institutional Review Board at the Zhongnan Hospital of Wuhan University. Written informed consent from the participants’ legal guardian/next of kin was not required to participate in this study in accordance with the national legislation and the institutional requirements. Written informed consent was not obtained from the minor(s)’ legal guardian/next of kin for the publication of any potentially identifiable images or data included in this article.

## Author contributions

ZL, ST and YC, designed the experiments; JL, YH, JX, HZ is responsible for information collection and data statistics; YC is responsible for writing the paper. All authors contributed to the article and approved the submitted version.
